# Sibling Influences on Trajectories of Maladaptive Behaviors in Autism

**DOI:** 10.3390/jcm11185349

**Published:** 2022-09-12

**Authors:** Nicole E. Rosen, Hillary K. Schiltz, Catherine Lord

**Affiliations:** Semel Institute for Neuroscience and Human Behavior, University of California, Los Angeles, CA 90095, USA

**Keywords:** siblings, maladaptive behaviors, autism, longitudinal, multi-informant, trajectories

## Abstract

Siblings play an important role in the behavioral trajectories of individuals with autism spectrum disorder (ASD). While having siblings has been associated with positive outcomes in ASD, including stronger adaptive functioning, social and non-verbal communication, and theory of mind, little is known about the impact of siblings on more negative outcomes, such as maladaptive behaviors. To address this gap, the present longitudinal study tested sibling predictors of trajectories of maladaptive behaviors (e.g., teacher- and parent-reported hyperactivity, irritability, and social withdrawal) from childhood through early adulthood among individuals with ASD and non-spectrum delays. Multilevel models revealed that, while the mere presence of a sibling did not impact maladaptive behavior trajectories apart from teacher-reported hyperactivity, the diagnostic profile of the sibling (e.g., emotional/behavioral disorder, ASD, medical condition) emerged as an important predictor. Specifically, although findings varied across teacher and parent reports, more hyperactivity and irritability across time was identified when the sibling had diagnoses of an emotional/behavioral disorder, ASD, and/or a medical condition. Overall, this study provides novel insight into the broader family-level factors that influence the presentation of maladaptive behaviors across time and across contexts.

## 1. Introduction

Siblings serve as models of behavior and have the potential to shape the developmental trajectories of people with autism spectrum disorder (ASD) due to frequent shared experiences and contact across the lifespan [1,2,3,4,5]. The sibling relationship may be particularly important for individuals with ASD, for whom challenges in peer interactions may necessitate greater reliance on sibling interactions to foster skill development [4,5,6]. In ASD, having siblings has been associated with better parent-reported adaptive functioning over time [7], social and non-verbal communication skills [8,9], and theory of mind abilities [10,11,12]. It is unknown, however, whether siblings impact maladaptive behaviors in ASD.

Behavioral modeling has been identified as an important learning mechanism within sibling relationships [1,13], particularly for neurodiverse sibling pairs [4,5,6]. Siblings can shape each other’s positive and negative behaviors through observation, imitation, and reinforcement [13,14]. That is, while these mechanisms of learning in sibling relationships provide the foundation for developing positive behaviors that build social and adaptive competencies, they also have the potential to breed aggression, hostility, and other maladaptive behaviors [1,13]. The presence of maladaptive behaviors in the sibling relationship has also been systematically linked to problems with peer interactions and relationships [14]. Moreover, maladaptive behaviors, including hyperactivity, irritability, social withdrawal, aggression, and other socially-inappropriate behaviors, can affect everyday functioning within multiple social environments, including at home and at school [15,16]. Maladaptive behaviors are particularly common in ASD—affecting over half of individuals with ASD [17,18,19,20]—and have been shown to significantly impact a range of short- and long-term outcomes, including academic achievement, social competence, and adult psychiatric functioning [16,21]. Therefore, it is important to understand factors that may exacerbate or ameliorate such maladaptive behaviors in ASD across multiple social contexts (i.e., home and school via parent and teacher reports); the presence of a sibling, serving as a behavioral model, may be one such factor.

While it might be that merely having a sibling affects the presentation and development of maladaptive behaviors in ASD, it may also be that the diagnostic characteristics of that sibling play a key role. Siblings of individuals with ASD are genetically at heightened risk for having ASD, subclinical ASD, maladaptive behaviors, and other psychiatric conditions themselves. Current estimates suggest that 10–20% of siblings of individuals with ASD will also have ASD themselves [22,23] compared to the 2.2% national US prevalence [24]. Of the remaining siblings of individuals with ASD who do not themselves meet the criteria for ASD, an additional 20–30% may, at some point, demonstrate other developmental atypicalities [25,26]. These diagnostic profiles may set the stage for social and emotional difficulties, aggression, and overall adjustment problems [6,27,28], leading to the modeling and reinforcement of maladaptive behaviors for the target individual with ASD [1,13,14]. 

In addition to modeling mechanisms of behavior, family processes may also play a role in shaping trajectories of maladaptive behaviors in ASD, especially when more than one child in the family experiences emotional, behavioral, and/or social challenges. Within families containing multiple children, caregiver attention and resources are necessarily divided amongst children. It may be that, when siblings experience maladaptive behaviors or other conditions, they not only require greater caregiver time (e.g., to manage such behaviors, identify and provide necessary supports/services, and carry out general parenting tasks) but may also be more affected by a lack of parental attention than children without such challenges [29,30]. Thus, heightened sibling-related stress and decreased availability of parents for everyday support and attention may exacerbate maladaptive behaviors in the sibling pair, especially in the home environment [31]. Given this potential family stress mechanism, caregivers may be even more sensitive to these processes and behaviors than reporters from other contexts (e.g., school).

Given the lifelong relationship between siblings, the impact of siblings on maladaptive behaviors may emerge and fluctuate across development, leading to differential trajectories of these behaviors over time. A growing body of literature indicates that maladaptive behaviors are not stagnant; rather, Rosen and colleagues [32] identified significant improvements in teacher- and parent-reported maladaptive behavior trajectories in ASD from ages 9 to 18, specifically in hyperactivity and irritability, and a slight worsening or plateauing of social withdrawal symptom trajectories. Consistent with the broader literature [15,33,34], individual differences in verbal intelligence quotient (VIQ) and ASD features in this sample were related to differential trajectories of maladaptive behaviors (e.g., greater cognitive impairment and ASD symptom severity are associated with more maladaptive behaviors and variable patterns of change over time) [32]. Apart from Rosen and colleagues [32], the majority of longitudinal research in this area relies solely on parent report; therefore, there is a need for continued investigation using multi-informant approaches (e.g., parent and teacher reports) to understand sibling effects across both time and context [32,35,36]. As such, while our understanding of the impact of various individual-level factors (e.g., cognitive ability, ASD symptom severity) on maladaptive behavior trajectories in ASD is expanding, little research has assessed broader family-level factors, such as siblings, and no research, to our knowledge, has assessed these factors from a multi-informant perspective. 

Although autism research demonstrates that siblings can promote positive outcomes across various domains of functioning, it is also important to determine whether having a sibling, and the diagnostic profile of that sibling, may negatively impact outcomes, such as the presence and development of maladaptive behaviors over time in ASD, using a multi-informant approach (e.g., teacher and parent report). In light of this need, the present study aimed to (1) examine the impact of the presence of a sibling on developmental trajectories of teacher- and parent-reported maladaptive behaviors (i.e., hyperactivity, irritability, and social withdrawal), and (2) assess the influence of the sibling diagnostic profile (i.e., sibling emotional/behavioral disorder, sibling ASD, sibling medical condition) on these trajectories among individuals with ASD or non-spectrum delays from ages 9 to 18.

## 2. Materials and Methods

### 2.1. Participants 

The sample for the present study was selected from an ongoing longitudinal study that originally recruited participants from the following sources: (1) children under age 3 years referred for possible ASD or other developmental delays to autism programs in North Carolina and Illinois (*n* = 213), and (2) children with ASD or other developmental delays diagnosed at early ages in Michigan who joined the study at approximately age 9 (*n* = 40) (see Anderson et al., 2014 for recruitment details [37]). 

The sample in the current study is identical to the sample used in the Rosen et al. study [32] that established the baseline trajectory models upon which this study builds. Of the original 253 participants enrolled in the longitudinal study, 165 were selected for the present study based on the availability of teacher- and parent-reported data between ages 9 to 18. A majority had more than one timepoint of teacher-reported data (77.6% completed two or more, 70.9% completed three or more, 63.6% completed four or more) and parent-reported data (86.1% completed two or more, 83% completed three or more, 80% completed four or more), with over five teacher-reported timepoints and over ten parent-reported timepoints available on average per participant. Compared to the current sample, participants not included in analyses due to missing data (either due to attrition or data collection issues) had lower autism symptom severity (*p* = 0.04) and more advanced caregiver education (*p* < 0.01) but did not differ on gender, recruitment site, diagnostic history (ever/never ASD), race, or VIQ. The sample was predominantly male (79.4%) and White (75.2%), and the majority of the sample was recruited from North Carolina.

Among the 165 participants in this study, 138 (83.6%) reported having at least one sibling (including full biological, half, step, and adopted siblings) with whom they lived during childhood. The age difference from their closest-age sibling was less than 9 years for nearly all participants with at least one sibling (98%); many were even closer in age (84.8% were within 5 years, 45.7% were within 3 years, and 39.1% were within 2 years). Thus, the current study utilized data beginning at age 9 given that approximately all (98%) participants with siblings were living with their closest-age sibling by this age. Related to birth order, 54.3% of participants were youngest children, 22.5% were middle children, and 23.2% were oldest children. Among participants with siblings, the number of siblings reported per participant included the following breakdown: 73 (52.9%) had one sibling, 35 (25.4%) had two siblings, 22 (15.9%) had three siblings, 3 (2.2%) had four siblings, and 5 (3.6%) had five siblings. Of the 138 closest-age siblings, half of whom were male, 70 (50.7%) had at least one parent-reported diagnosis – 62 (44.9%) had an emotional or behavioral disorder, 22 (15.9%) had ASD, and 16 (11.6%) had a medical condition. The 138 participants with at least one sibling did not differ from the 27 participants without siblings in gender, diagnostic history (ever/never ASD), autism symptom severity, or VIQ. However, samples differed in race (more Black than White participants did not have siblings; *p* = 0.01), caregiver education (caregivers of participants without siblings had more education; *p* < 0.01), and recruitment site (participants without siblings were more likely to be recruited from North Carolina; *p* < 0.01).

### 2.2. Procedures

This research received institutional review board (IRB) approvals at various institutions across the duration of the study. Informed consent was obtained from all participating caregivers, teachers, and individuals themselves whenever possible. Caregivers, teachers, and participants (if able) completed various questionnaire packets via mail or during in-person visits multiple times throughout the study (a maximum of 16 times spaced on average 6 months apart between ages 9–18). Rates of measure completion at the different timepoints were primarily affected by (1) whether the measures were completed as part of an in-person assessment visit or as part of a mailed packet (greater completion for in-person visits), and (2) the proximity (in time) of the measure timepoint to an in-person assessment visit (greater completion during timepoints immediately before and after in-person visits). Additionally, as is common in longitudinal studies, attrition due to families moving or changing contact information has affected the sample, resulting in fewer completed measures at age 18 than at age 9. Additional measures, including diagnostic and cognitive assessments, were completed during in-person assessments (except teachers) at two timepoints during this study’s duration (9–18 years of age). Most participants had completed at least one in-person assessment by age 9 (mean (*M*) years = 9.98, standard deviation (*SD*) = 0.89), with additional in-person assessments at approximately ages 18 (*M* = 19.04, *SD* = 1.2) and 26 (*M* = 25.97, *SD* = 1.4). All visits and assessments were provided free of charge, and feedback on the testing results was provided. Diagnostic decisions during in-person visits were made by the research team, all of whom were research reliable on the relevant measures, and confirmed by an expert clinician panel. Teachers were mailed a small gift as compensation upon completing the questionnaires.

### 2.3. Measures

*Autism Features.* Participants’ Autism Diagnostic Observation Schedule-Calibrated Severity Scores (ADOS-CSS; [38,39,40]) at age 9 (if unavailable, then from later years) were used to describe autism symptom severity. At each in-person assessment at approximately ages 9, 18, and 26 years old, participants were administered the ADOS. The ADOS-CSS can be used to compare ASD features across individuals of different developmental levels, with scores ranging from 1 to 10, with higher scores indicating higher levels of autism-spectrum-related features observed during the ADOS.

*Cognitive Abilities.* Cognitive assessments were administered based on the following standard hierarchy: Wechsler Abbreviated Scale of Intelligence (WASI; [41]), Wechsler Intelligence Scale for Children (WISC-III; [42]), and Differential Abilities Scale (DAS; [43,44]); age 9 assessment results (if unavailable, from later years) were used in the present study. VIQ was selected to be consistent with existing literature. 

*Sibling Information.* Parents reported information about the participants’ siblings, including the number of siblings in the family and other standard demographic information (e.g., age, gender, race, caregiver education, etc.) about the closest-age sibling. Additionally, parents provided information about the closest-age sibling’s diagnostic profile by responding to the question “Has the sibling EVER been diagnosed with a medical or psychiatric condition? If YES, please write the condition” on the demographic form. Diagnoses were then grouped into three categories: (1) emotional/behavioral conditions broadly, including attention-deficit/hyperactivity disorder, depression, anxiety, oppositional defiant disorder, bipolar disorder, ASD, obsessive-compulsive disorder, and Tourette’s disorder; (2) ASD specifically; and (3) medical conditions, including epilepsy, cerebral palsy, cancer, juvenile rheumatoid arthritis, sensory impairments (e.g., hearing and vision), cardiovascular conditions, blood disorders, traumatic brain injury, and autoimmune diseases. 

*Maladaptive Behaviors.* Maladaptive behaviors were assessed via the Aberrant Behavior Checklist (ABC; [45]), a questionnaire designed to assess Hyperactivity, Irritability, Social Withdrawal/Lethargy, Stereotypic Behavior, and Inappropriate Speech in people with developmental disabilities. Teachers and parents completed the ABC yearly from ages 9 to 18 (see Table S1 for sample sizes at each age grouping). A total of 58 items are scored on a four-point Likert scale ranging from (0) “not at all a problem” to (3) “the problem is severe in degree”. The Stereotypic Behavior and Inappropriate Speech subscales were excluded from the present analyses due to the significant overlap of these items with core ASD features [33,46]. The ABC is commonly used in ASD research due to its psychometric strengths, including moderate-to-high reliability [47], good validity [45,48], and strong psychometric performance among individuals with ASD [49,50,51]. ABC teacher and parent reports in the current sample show moderate interrater agreement for hyperactivity (*r* = 0.38–0.48) and irritability (*r* = 0.40–0.45), and low interrater agreement for social withdrawal (*r* = 0.10–0.25). 

### 2.4. Statistical Analyses 

ABC-Hyperactivity, -Irritability, and -Social Withdrawal variables were screened for normal distributions and extreme values and were determined to be within normal limits. The current analyses are built on the multilevel models established in Rosen et al. [32]. As described in Rosen et al. [32], we utilized multilevel modeling and maximum likelihood estimation with robust standard errors (MLR) in Mplus v. 8.1 [52] to examine changes in teacher- and parent-reported ABC-Hyperactivity, -Irritability, and -Social Withdrawal from ages 9 to 18. These initial trajectory models (for results of each step of these analyses, see Rosen et al. [32]) were built by (1) testing the appropriateness of capturing between-participant and between-recruitment site effects (i.e., assessing the structure of the data with respect to age/time (level 1), individual (level 2), and site (level 3) [53,54]), (2) assessing the rate of change of maladaptive behavior outcomes (i.e., hyperactivity, irritability, and social withdrawal) as a function of participant age (first as a fixed effect and then adding the random effect), and (3) examining multiple demographic factors (e.g., gender, race, and caregiver education), individual characteristics (e.g., ADOS-CSS and VIQ), and other potential covariates (e.g., consistency in reporter) in relation to the intercept and slope of all models. Therefore, relevant covariates available for both teacher and parent report were selected based on empirical evidence (see Table 1) and were included in all sibling analyses described in this paper; relevant covariates were all mean-centered for ease of interpretability (i.e., all graphs and analyses presented here are interpreted at the mean of the relevant covariates).

Building on the model described above, we first estimated intercept and slope of hyperactivity, irritability, and social withdrawal from ages 9 to 18 while controlling for relevant covariates that were empirically identified in our previous study (see Table 1) [32]. Next, in order to examine the effect of siblings above and beyond relevant individual-level factors, binary sibling variables were individually added to the models, including the presence of a sibling (yes/no) and, for those with siblings, whether the sibling has an emotional or behavioral disorder (yes/no), ASD (yes/no), and/or a medical condition (yes/no). Each sibling variable was tested individually as a predictor of the intercept or slope (i.e., cross-level interactions with age) in the teacher- and parent-reported ABC-Hyperactivity, ABC-Irritability, and ABC-Social Withdrawal models. Significant effects were plotted for visualization purposes; the marginal effects of sibling variables are plotted at the mean of relevant covariates for that particular model (see Table 1).

To orient the reader to the organizational structure of our results, we first present teacher report followed by parent report within each set of analyses. Generally, we discuss our outcomes in the following order: Hyperactivity, then Irritability, and Social Withdrawal last. First, we present results of the ABC-Hyperactivity, -Irritability, and -Social Withdrawal trajectory models, controlling for relevant covariates (i.e., at the mean of the covariates; see Table 1). Next, significant findings from the presence of a sibling analyses are described, followed by a summary of nonsignificant findings. Lastly, sibling diagnostic predictor analyses are reported for each diagnosis (i.e., sibling emotional/behavioral disorder, sibling ASD, and sibling medical condition); significant findings are presented first, followed by a summary of nonsignificant findings.

## 3. Results

### 3.1. ABC Trajectory Models

*Hyperactivity.* After controlling for the effects of VIQ, ADOS-CSS, and reporter consistency (see Table 1) on intercept and slope (i.e., at the mean of these variables), the teacher-reported ABC-Hyperactivity model revealed an intercept of 13.35 (*p* < 0.01) and a significant decline in participant hyperactivity from ages 9 to 18 (*B* = −0.52, *p* < 0.01). Similarly, in the parent-reported ABC-Hyperactivity model, after controlling for the effects of VIQ (see Table 1) on intercept and slope, the model revealed an intercept of 14.24 (*p* < 0.01) and a significant decline in participant hyperactivity from ages 9 to 18 (*B* = −0.67, *p* < 0.01).

*Irritability.* After controlling for the effects of VIQ and ADOS-CSS (see Table 1) on intercept and slope, the teacher-reported ABC-Irritability model revealed an intercept of 9.20 (*p* < 0.01) and a nonsignificant decline in participant irritability from ages 9 to 18 (*B* = −0.21, *p* = 0.14). In the parent-reported ABC-Irritability model, after controlling for the effects of VIQ (see Table 1) on intercept and slope, the model revealed an intercept of 9.44 (*p* < 0.01) and a significant decline in participant irritability from ages 9 to 18 (*B* = −0.27, *p* < 0.01).

*Social Withdrawal.* After controlling for the effects of VIQ and ADOS-CSS (see Table 1) on intercept and slope, the teacher-reported ABC-Social Withdrawal model revealed an intercept of 9.63 (*p* < 0.01) and a nonsignificant slope of social withdrawal from ages 9 to 18 (*B* = 0.10, *p* = 0.54). Similarly, the parent-reported ABC-Social Withdrawal model revealed an intercept of 7.60 (*p* < 0.01) and a nonsignificant slope (*B* = 0.06, *p* = 0.44) after controlling for the effects of VIQ and ADOS-CSS (see Table 1) on participant social withdrawal across time. 

### 3.2. Presence of a Sibling Analyses 

**Figure 1:**
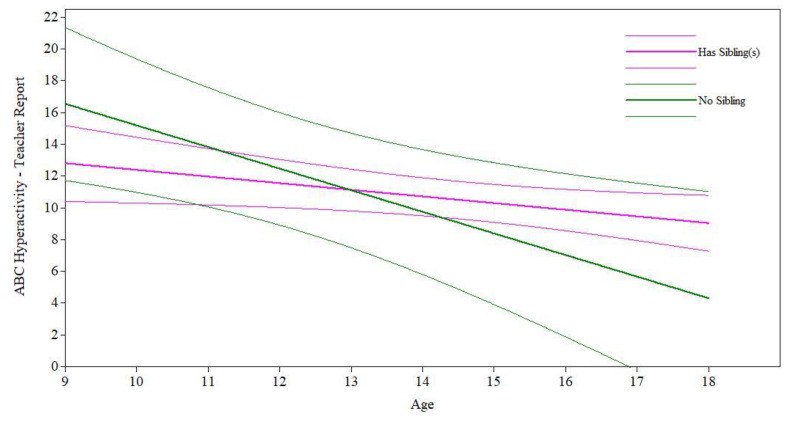
Presence of a sibling on maladaptive behavior trajectories.

The presence of a sibling had a nonsignificant effect on the teacher-reported ABC-Hyperactivity intercept (*B* = −3.75, *p* = 0.18) and a marginal effect on slope (*B* = 0.94, *p* = 0.09) after controlling for relevant covariates (see Table 1), such that participants had similar levels of teacher-reported hyperactivity at age 9 but participants with siblings improved marginally less over time in hyperactivity than participants without siblings (see Figure 1). The presence of a sibling did not have a significant effect on the intercept or slope of ABC-Irritability or ABC-Social Withdrawal as reported by teachers or parents after controlling for relevant covariates (see Table 1), nor on the intercept or slope of ABC-Hyperactivity as reported by parents (see Table 2). This suggests that, according to both teacher and parent reports, participants with and without siblings had similar levels of hyperactivity (only parent-reported), irritability, and social withdrawal at age 9 and experienced similar changes in symptoms over time from ages 9 to 18. 

### 3.3. Sibling Diagnostic Predictors Analyses 

**Figure 2:**
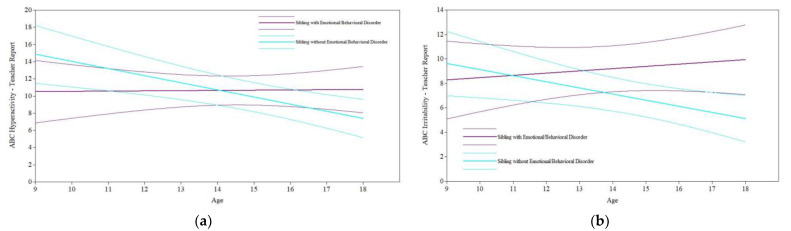
Sibling emotional/behavioral disorder on maladaptive behavior trajectories. (**a**) Sibling emotional/behavioral disorder on teacher-reported ABC-Hyperactivity. (**b**) Sibling emotional/behavioral disorder on teacher-reported ABC-Irritability.

*Sibling Emotional/Behavioral Disorder.* Among participants with siblings, controlling for relevant covariates (see Table 1), having a sibling with an emotional or behavioral disorder had a significant and marginal effect, respectively, on participants’ slope of teacher-reported hyperactivity (*B* = 0.86, *p* = 0.03; see Figure 2a) and irritability (*B* = 0.68, *p* = 0.06; see Figure 2b); however, no significant effects on the intercepts (hyperactivity: *B* = −4.34, *p* = 0.10; irritability: *B* = −1.34, *p* = 0.55) were found (see Table 2). Thus, according to teacher reports, participants with siblings with an emotional or behavioral disorder experienced significantly less improvement in hyperactivity over time and marginally less improvement in irritability over time, even a slight worsening of symptoms, than participants with siblings who did not have an emotional or behavioral disorder, although their initial levels of hyperactivity and irritability did not differ. Having a sibling with an emotional or behavioral disorder did not significantly impact the teacher-reported social withdrawal intercept or slope after controlling for relevant covariates (see Table 1 and Table 2). According to parent reports, controlling for relevant covariates (see Table 1), having a sibling with an emotional or behavioral disorder did not have significant effects on the intercepts or slopes for hyperactivity, irritability, or social withdrawal (see Table 2). 

**Figure 3:**
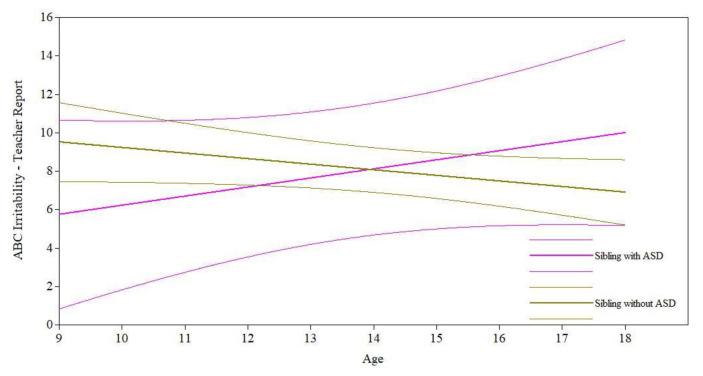
Sibling ASD diagnosis on maladaptive behavior trajectories.

*Sibling ASD.* Controlling for the effects of relevant covariates (see Table 1), having a sibling with an ASD diagnosis had a marginal effect on participants’ teacher-reported irritability slope (*B* = 0.76, *p* = 0.08; see Figure 3) but a nonsignificant effect on the irritability intercept (*B* = −3.77, *p* = 0.18). While participants had similar levels of irritability at age 9, participants with siblings with an ASD diagnosis experienced marginally less improvement over time in irritability than participants with siblings who did not have an ASD diagnosis. However, for parent-reported ABC-Irritability and for both teacher- and parent-reported ABC-Hyperactivity and ABC-Social Withdrawal, having a sibling with ASD did not significantly predict intercept or slope after controlling for relevant covariates (see Table 1). This indicates that participants with siblings with and without ASD had similar levels of hyperactivity, irritability, and social withdrawal at age 9 and experienced similar changes, apart from teacher-reported irritability, in these symptoms over time. 

**Figure 4:**
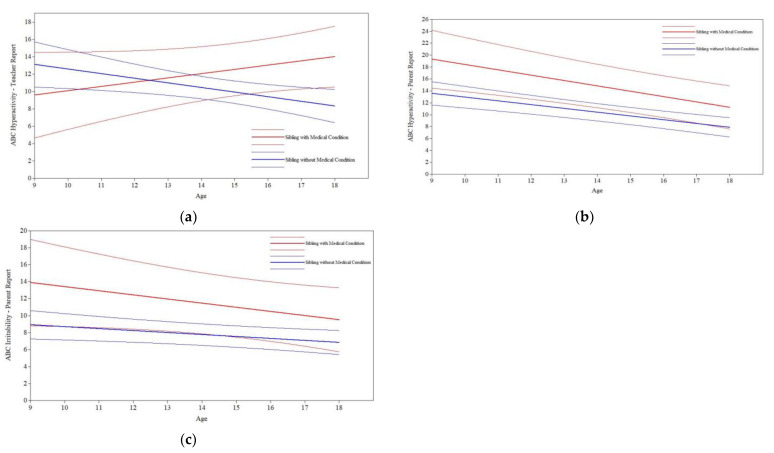
Sibling medical condition on maladaptive behavior trajectories. (**a**) Sibling medical condition on teacher-reported ABC-Hyperactivity. (**b**) Sibling medical condition on parent-reported ABC-Hyperactivity. (**c**) Sibling medical condition on parent-reported ABC-Irritability.

*Sibling Medical Condition.* Having a sibling with a medical condition had a significant effect on the participants’ teacher-reported hyperactivity slope (*B* = 1.02, *p* < 0.01; see Figure 4a) but a nonsignificant effect on the intercept (*B* = −3.52, *p* = 0.22) after controlling for relevant covariates (see Table 1). While participants had similar levels of teacher-reported hyperactivity at age 9, participants with siblings who had a medical condition experienced increasing teacher-reported hyperactivity over time compared to declining teacher-reported hyperactivity among participants with siblings who did not have a medical condition. Unlike teacher-reported hyperactivity trajectories, having a sibling with a medical condition had a significant effect on participants’ parent-reported hyperactivity intercept (*B* = 5.74, *p* = 0.03; see Figure 4b) but a nonsignificant effect on slope (*B* = −0.27, *p* = 0.33), controlling for relevant covariates (see Table 1). Thus, while participants with siblings with a medical condition had significantly higher levels of parent-reported hyperactivity at age 9 than participants with siblings who did not have a medical condition, both participant groups experienced similar rates of improvement in parent-reported hyperactivity over time.

Having a sibling with a medical condition had a nonsignificant effect on the participants’ teacher-reported ABC-Irritability intercept and slope after controlling for relevant covariates (see Table 1). In contrast, for parent-reported ABC-Irritability trajectories, having a sibling with a medical condition had a marginal effect on the participants’ intercept (*B* = 4.98, *p* = 0.07; see Figure 4c) and a nonsignificant effect on slope (*B* = −0.26, *p* = 0.40), controlling for relevant covariates (see Table 1). Thus, while participants with siblings with a medical condition had greater parent-reported irritability at age 9 than participants with siblings without a medical condition, the groups did not differ in their rates of improvement in irritability from ages 9 to 18. Lastly, for social withdrawal, after controlling for the effects of covariates (see Table 1), having a sibling with a medical condition had nonsignificant effects on participants’ teacher-reported and parent-reported ABC-Social Withdrawal intercepts and slopes.

## 4. Discussion

The present study extends the field’s understanding of predictors of maladaptive behavior development in ASD, building from individual-level predictors, such as VIQ and ASD features [15,32,33,55], to broader family-level factors. In particular, these findings provide novel insight, from both teacher and parent perspectives, into the influence of siblings and the sibling diagnostic profile on maladaptive behavior trajectories among individuals with ASD and non-ASD developmental delays from ages 9 to 18. Overall, while effects of sibling diagnosis on hyperactivity and irritability in ASD emerged, particularly according to teacher reports, the results indicate a less clear impact of the mere presence of siblings on maladaptive behaviors. 

In contrast to previous research examining “positive” outcomes, the presence of a sibling did not have a robust influence on maladaptive behavior trajectories in the present study. More specifically, prior studies have reported that the presence of a sibling is linked with better adaptive functioning, communication, and perspective taking in ASD [7,8,9,11]. In contrast, in the current study, the presence of a sibling only had a marginal effect on the slope of one teacher-reported outcome (hyperactivity). Perhaps, the sibling relationship lends itself well to learning and promoting skills that are likely to be practiced and modeled frequently (e.g., engaging in social communication, doing tasks independently), but not behaviors that may be less likely to occur (especially for siblings without diagnoses). The lack of a robust effect of the presence of a sibling on maladaptive behaviors is welcomed as it suggests that having a sibling may be more important for promoting “positive” than exacerbating “negative” behaviors and skills, even if no buffering effect was found (i.e., having a sibling was not protective against maladaptive behaviors in ASD).

Although having a sibling had little effect on maladaptive behaviors in ASD, the siblings’ diagnostic characteristics emerged as important factors. Less improvement was observed in teacher-reported hyperactivity and irritability from ages 9 to 18 for participants with ASD or non-spectrum delays whose siblings also had diagnoses themselves (i.e., with an emotional/behavioral disorder, ASD, and/or a medical condition) compared to participants whose siblings did not. This pattern is consistent with the principles of behavioral modeling [56,57,58]. Since self-regulatory capacity is often fostered through environmental supports, such as observing adults and peers (including siblings) engaging in their own self-regulatory strategies [15,59,60,61], inappropriate behavioral modeling may have reinforced maladaptive behaviors in participants over time [55], especially during formative periods (i.e., childhood and adolescence) [62,63]. 

It is important to note that sibling effects may be bidirectional within the dyad. Furthermore, siblings with diagnoses may not have coping skills to effectively diffuse maladaptive situations, thereby resulting in heightened dysregulated behaviors across development [64,65,66]. These challenges within the sibling dyad may also be exacerbated by family stress mechanisms such that families parenting multiple children with behavioral difficulties or requiring significant support may set the stage for worsening (or less improvement) in problems over time due to depleted parental resources [31,67,68]. 

Across the different sibling diagnoses, while the presence of an emotional or behavioral disorder and/or ASD in the sibling had a more consistent effect on teacher-reported maladaptive behaviors (i.e., hyperactivity and irritability), the presence of a medical condition in siblings was a more consistent factor impacting parent-reported maladaptive behaviors. These differing patterns may suggest that family-stress mechanisms could operate more for medical conditions, while behavioral modeling mechanisms could operate more for emotional/behavioral conditions or ASD. Existing research also supports this theory given that parents of children with medical conditions report lower overall quality of life, greater perceived burden, and increased stress than parents of children with emotional or behavioral conditions [69,70]; teachers report the greatest levels of stress when teaching students with emotional or behavioral problems [71]. Alternatively, the differing pattern of sibling diagnostic effects based on parent and teacher reports may be due to differential manifestations of behaviors across contexts (e.g., in school versus at home) or differing sensitivity of reporters to these particular behaviors (e.g., teachers have many comparison children without difficulties, while parents may be influenced by raising multiple children with behavioral challenges), thus highlighting the importance of the multi-informant approach to better understand the full behavioral profile of individuals with ASD.

The presence of a sibling and the diagnostic profile of the sibling did not impact social withdrawal trajectories. Consistent with previous research [32,33], the present study seems to support a relatively unchanging pattern of social withdrawal across time, perhaps even in the face of different family/environmental conditions (i.e., lack of sibling effects). The lack of a sibling effect on social withdrawal may be explained, in part, by potential differential effects of the behavioral modeling processes involving externalizing (e.g., hyperactivity, irritability) versus internalizing (e.g., social withdrawal) symptoms. Symptoms that are more visible (e.g., externalizing) may be more sensitive to the effects of behavioral modeling than behaviors that are more difficult to readily identify (e.g., internalizing) [65,66]. Externalizing behaviors are also found to be more closely tied with family functioning (e.g., fewer family social activities and outings, weaker sibling relationships, fewer opportunities for play, and overall greater stress within the family unit) [30,72] than internalizing symptoms, which aligns with the family factors examined in our study (i.e., sibling diagnosis) having more of an impact on hyperactivity and irritability than on social withdrawal. 

The present study has important implications for clinical practice. Given the significant impact of sibling diagnoses on hyperactivity and irritability trajectories in ASD, providing intervention services to both siblings in a dyad may be helpful to maximize opportunities for appropriate behavioral modeling. This is particularly relevant for people with ASD given their sometimes limited opportunities to engage with peers and learn social–behavioral skills [8]; as such, the accessibility of siblings [73,74] makes them an important source of modeling to supplement skill development [8]. Relatedly, providing support to siblings regarding strategies to improve their interactions (e.g., conflict resolution, coping skills, play skills, etc.) may also be a critical intervention target during this developmental period given the intertwined nature of behavior and development of siblings. Overall, the results from this study suggest that the impact of the broader family unit on the behavioral functioning of individuals with ASD and non-ASD developmental delays should be considered, in addition to previously identified individual-level factors [15,32,33,55], when conceptualizing an individual’s service plan and goals.

In addition to the strengths of this study, including the novel use of sibling diagnoses in ASD behavioral trajectory analyses, the multi-informant approach, and the longitudinal design, there are limitations that provide directions for future research. Sibling diagnoses were reported by parents, while participant diagnoses were comprehensively assessed and given by the research team; thus, future studies may wish to conduct diagnostic testing for both participants and their siblings to ensure consistency in diagnostic practices. As described previously [37,75], given that most participants in the present study were recruited and diagnosed with ASD or non-ASD developmental delays before age 3, the findings may not be representative of children first diagnosed at later ages. Additionally, as is common in longitudinal studies, attrition has affected the sample, resulting in fewer participants from marginalized communities. Furthermore, as is also common in longitudinal research, the present analyses should be considered within the context of variable participant engagement observed across the duration of the decades-long study (i.e., participating at certain timepoints, disengaging from the study for a few years, and re-engaging with the study a few years later). The sample size is also relatively small, which not only tempers our confidence in these parameter estimates but also limits our ability to assess gender or cultural differences. The existing literature on sibling gender effects is mixed, such that having a male closest-age sibling has been associated with stronger adaptive skill growth over time for individuals with ASD [7], although female siblings have been noted to show greater caregiving, companionship, and positive affect in sibling relationships [76]; thus, future research with larger samples is needed to explore the role of sibling gender on maladaptive behavior trajectories. Additionally, the current study only examined sibling-related family effects; other studies are needed to test whether other family factors (e.g., parenting stress, family cohesion, etc.) may also impact trajectories of maladaptive behaviors in ASD. Lastly, given the limitations of the ABC as an informant-report measure, future studies may benefit from replication of the present study with an in-depth clinical interview to evaluate consistency in these findings across multiple methodologies.

## 5. Conclusions

The present longitudinal study examined sibling predictors of trajectories of teacher- and parent-reported maladaptive behaviors from childhood through early adulthood among individuals with ASD and non-spectrum delays. The findings suggest that, while the mere presence of a sibling did not impact maladaptive behavior trajectories apart from teacher-reported hyperactivity, the diagnostic profile of the sibling emerged as an important predictor of hyperactivity and irritability from ages 9 to 18. While the findings varied across teacher and parent reports, individuals with ASD or non-ASD delays with siblings who themselves had diagnoses of an emotional/behavioral disorder, ASD, and/or a medical condition experienced higher levels of and/or slower improvement in hyperactivity and irritability across time. Overall, this study provides novel insight into the broader family-level factors that influence the presentation of maladaptive behaviors across time and across contexts. 

## Figures and Tables

**Table 1:** Significant covariates for teacher- and parent-reported ABC-Hyperactivity, -Irritability, and -Social Withdrawal.

	Hyperactivity	Irritability	Social Withdrawal
Teacher Report	VIQ, ADOS-CSS, Reporter Consistency	VIQ, ADOS-CSS	VIQ, ADOS-CSS
Parent Report	VIQ	VIQ	VIQ, ADOS-CSS

Note: please see Rosen et al. [32] for further information related to covariate selection; VIQ = verbal intelligence quotient; ADOS-CSS = Autism Diagnostic Observation Schedule-Calibrated Severity Score

**Table 2:** Sibling effects on ABC trajectories.

	Hyperactivity				Irritability				Social Withdrawal			
	Teacher		Parent		Teacher		Parent		Teacher		Parent	
Predictors	*B* on Intercept	*B* on Slope	*B* on Intercept	*B* on Slope	*B* on Intercept	*B* on Slope	*B* on Intercept	*B* on Slope	*B* on Intercept	*B* on Slope	*B* on Intercept	*B* on Slope
Presence of a Sibling	−3.75	0.94 ^	−0.08	0.00	−1.01	0.37	0.15	0.23	−1.58	0.25	−3.03	0.32
Sibling Dx: Emotional/Behavioral Disorder	−4.34	0.86 *	0.12	−0.05	−1.34	0.68 ^	1.01	0.04	−0.86	0.30	−0.26	−0.10
Sibling Dx: ASD	−3.80	0.62	−0.26	0.01	−3.77	0.76 ^	0.69	0.09	0.96	−0.17	−1.30	−0.21
Sibling Dx: Medical Condition	−3.52	1.02 *	5.74 *	−0.27	0.68	0.32	4.98 ^	−0.26	−2.01	0.56	0.80	0.01

Note. * significant effect at alpha of 0.05; ^ marginal effect at alpha of 0.10; Dx = diagnosis

## Data Availability

Not applicable.

## Supplementary Materials

The following supporting information can be downloaded at: https://www.mdpi.com/article/10.3390/jcm11185349/s1, Table S1: Teacher- and parent-reported ABC sample sizes across age groupings.

## References

[1] Cicirelli V. (2013). Sibling Relationships Across the Life Span.

[2] Dunn J. (2007). Siblings and Socialization. Handbook of Socialization: Theory and Research.

[3] Lamb M.E., Sutton-Smith B., Sutton-Smith B., Lamb M.E. (2014). Sibling Relationships: Their Nature and Significance Across the Lifespan.

[4] Ferraioli S.J., Hansford A., Harris S.L. (2012). Benefits of Including Siblings in the Treatment of Autism Spectrum Disorders. Cogn. Behav. Pract..

[5] Knott F., Lewis C., Williams T. (2007). Sibling Interaction of Children with Autism: Development Over 12 Months. J. Autism Dev. Disord..

[6] Orsmond G.I., Seltzer M.M. (2007). Siblings of Individuals with Autism Spectrum Disorders across the Life Course. Ment. Retard. Dev. Disabil. Res. Rev..

[7] Rosen N.E., McCauley J.B., Lord C. (2022). Influence of Siblings on Adaptive Behavior Trajectories in Autism Spectrum Disorder. Autism.

[8] Ben-Itzchak E., Zukerman G., Zachor D.A. (2016). Having Older Siblings Is Associated with Less Severe Social Communication Symptoms in Young Children with Autism Spectrum Disorder. J. Abnorm. Child. Psychol..

[9] Ben-Itzchak E., Nachshon N., Zachor D.A. (2019). Having Siblings Is Associated with Better Social Functioning in Autism Spectrum Disorder. J. Abnorm. Child. Psychol..

[10] Matthews N.L., Goldberg W.A., Lukowski A.F. (2013). Theory of Mind in Children with Autism Spectrum Disorder: Do Siblings Matter?. Autism Res..

[11] Matthews N.L., Goldberg W.A. (2018). Theory of Mind in Children with and without Autism Spectrum Disorder: Associations with the Sibling Constellation. Autism.

[12] O’Brien K., Slaughter V., Peterson C.C. (2011). Sibling Influences on Theory of Mind Development for Children with ASD. J. Child. Psychol. Psychiatry.

[13] Whiteman S.D., McHale S.M., Soli A. (2011). Theoretical Perspectives on Sibling Relationships. J. Fam. Theory Rev..

[14] Dunn J. (1988). Sibling Influences on Childhood Development. J. Child. Psychol. Psychiatry.

[15] Shattuck P.T., Seltzer M.M., Greenberg J.S., Orsmond G.I., Bolt D., Kring S., Lounds J., Lord C. (2007). Change in Autism Symptoms and Maladaptive Behaviors in Adolescents and Adults with an Autism Spectrum Disorder. J. Autism Dev. Disord..

[16] De Bildt A., Sytema S., Kraijer D., Sparrow S., Minderaa R. (2005). Adaptive Functioning and Behaviour Problems in Relation to Level of Education in Children and Adolescents with Intellectual Disability. J. Intellect. Disabil. Res..

[17] Baghdadli A., Pascal C., Grisi S., Aussilloux C. (2003). Risk Factors for Self-Injurious Behaviours among 222 Young Children with Autistic Disorders. J. Intellect. Disabil. Res..

[18] Holden B., Gitlesen J.P. (2006). A Total Population Study of Challenging Behaviour in the County of Hedmark, Norway: Prevalence, and Risk Markers. Res. Dev. Disabil..

[19] Matson J.L., Wilkins J., Macken J. (2008). The Relationship of Challenging Behaviors to Severity and Symptoms of Autism Spectrum Disorders. J. Ment. Health Res. Intellect. Disabil..

[20] Simonoff E., Pickles A., Charman T., Chandler S., Loucas T., Baird G. (2008). Psychiatric Disorders in Children with Autism Spectrum Disorders: Prevalence, Comorbidity, and Associated Factors in a Population-Derived Sample. J. Am. Acad. Child. Adolesc. Psychiatry.

[21] Kim J.A., Szatmari P., Bryson S.E., Streiner D.L., Wilson F.J. (2000). The Prevalence of Anxiety and Mood Problems among Children with Autism and Asperger Syndrome. Autism.

[22] Hansen S.N., Schendel D.E., Francis R.W., Windham G.C., Bresnahan M., Levine S.Z., Reichenberg A., Gissler M., Kodesh A., Bai D. (2019). Recurrence Risk of Autism in Siblings and Cousins: A Multinational, Population-Based Study. J. Am. Acad. Child. Adolesc. Psychiatry.

[23] Ozonoff S., Young G.S., Carter A., Messinger D., Yirmiya N., Zwaigenbaum L., Bryson S., Carver L.J., Constantino J.N., Dobkins K. (2011). Recurrence Risk for Autism Spectrum Disorders: A Baby Siblings Research Consortium Study. Pediatrics.

[24] Maenner M.J., Shaw K.A., Bakian A.V., Bilder D.A., Durkin M.S., Esler A., Furnier S.M., Hallas L., Hall-Lande J., Hudson A. (2021). Prevalence and Characteristics of Autism Spectrum Disorder Among Children Aged 8 Years—Autism and Developmental Disabilities Monitoring Network, 11 Sites, United States, 2018. MMWR Surveill. Summ..

[25] Charman T., Young G.S., Brian J., Carter A., Carver L.J., Chawarska K., Curtin S., Dobkins K., Elsabbagh M., Georgiades S. (2017). Non-ASD Outcomes at 36 Months in Siblings at Familial Risk for Autism Spectrum Disorder (ASD): A Baby Siblings Research Consortium (BSRC) Study. Autism Res..

[26] Messinger D., Young G.S., Ozonoff S., Dobkins K., Carter A., Zwaigenbaum L., Landa R.J., Charman T., Stone W.L., Constantino J.N. (2013). Beyond Autism: A Baby Siblings Research Consortium Study of High-Risk Children at Three Years of Age. J. Am. Acad. Child. Adolesc. Psychiatry.

[27] Meyer K.A., Ingersoll B., Hambrick D.Z. (2011). Factors Influencing Adjustment in Siblings of Children with Autism Spectrum Disorders. Res. Autism Spectr. Disord..

[28] Orsmond G.I., Seltzer M.M. (2009). Adolescent Siblings of Individuals with an Autism Spectrum Disorder: Testing a Diathesis-Stress Model of Sibling Well-Being. J. Autism Dev. Disord..

[29] Dumas J.E., Wolf L.C., Fisman S.N., Culligan A. (1991). Parenting Stress, Child Behavior Problems, and Dysphoria in Parents of Children with Autism, down Syndrome, Behavior Disorders, and Normal Development. Exceptionality.

[30] Sikora D., Moran E., Orlich F., Hall T.A., Kovacs E.A., Delahaye J., Clemons T.E., Kuhlthau K. (2013). The Relationship between Family Functioning and Behavior Problems in Children with Autism Spectrum Disorders. Res. Autism Spectr. Disord..

[31] Kirchhofer S.M., Orm S., Haukeland Y.B., Fredriksen T., Wakefield C.E., Fjermestad K.W. (2022). A Systematic Review of Social Support for Siblings of Children with Neurodevelopmental Disorders. Res. Dev. Disabil..

[32] Rosen N.E., Schiltz H.K., Lord C. (2022). Teacher- and Parent-Reported Trajectories of Challenging Behavior Among Individuals with Autism. medRxiv.

[33] Anderson D.K., Maye M.P., Lord C. (2011). Changes in Maladaptive Behaviors from Midchildhood to Young Adulthood in Autism Spectrum Disorder. Am. J. Intellect. Dev. Disabil..

[34] Stringer D., Kent R., Briskman J., Lukito S., Charman T., Baird G., Lord C., Pickles A., Simonoff E. (2020). Trajectories of Emotional and Behavioral Problems from Childhood to Early Adult Life. Autism.

[35] Dickson K.S., Suhrheinrich J., Rieth S.R., Stahmer A.C. (2018). Parent and Teacher Concordance of Child Outcomes for Youth with Autism Spectrum Disorder. J. Autism Dev. Disord..

[36] De Los Reyes A., Kazdin A.E. (2005). Informant Discrepancies in the Assessment of Childhood Psychopathology: A Critical Review, Theoretical Framework, and Recommendations for Further Study. Psychol. Bull..

[37] Anderson D.K., Liang J.W., Lord C. (2014). Predicting Young Adult Outcome among More and Less Cognitively Able Individuals with Autism Spectrum Disorders. J. Child. Psychol. Psychiatry.

[38] Lord C., Risi S., Lambrecht L., Cook E.H., Leventhal B.L., DiLavore P.C., Pickles A., Rutter M. (2000). The Autism Diagnostic Observation Schedule-Generic: A Standard Measure of Social and Communication Deficits Associated with the Spectrum of Autism. J. Autism Dev. Disord..

[39] Lord C., Rutter M., DiLavore P., Risi S., Gotham K., Bishop S. (2012). Autism Diagnostic Observation Schedule–2nd Edition (ADOS-2).

[40] Gotham K., Pickles A., Lord C. (2009). Standardizing ADOS Scores for a Measure of Severity in Autism Spectrum Disorders. J. Autism Dev. Disord..

[41] Wechsler D. (1999). The Wechsler Abbreviated Scale for Intelligence.

[42] Wechsler D. (1991). Weschler Intelligence Scale for Children: Third Edition Manual.

[43] Elliot C.D. (1990). The Nature and Structure of Children’s Abilities: Evidence From the Differential Ability Scales. J. Psychoeduc. Assess..

[44] Elliott C.D. (2007). Differential Ability Scales.

[45] Aman M.G., Singh N.N., Stewart A.W., Field C.J. (1985). The Aberrant Behavior Checklist: A Behavior Rating Scale for the Assessment of Treatment Effects. Am. J. Ment. Defic..

[46] Fok M., Bal V.H. (2019). Differences in Profiles of Emotional Behavioral Problems across Instruments in Verbal versus Minimally Verbal Children with Autism Spectrum Disorder. Autism Res..

[47] Aman M.G., Richmond G., Stewart A.W., Bell J.C., Kissel R.C. (1987). The Aberrant Behavior Checklist: Factor Structure and the Effect of Subject Variables in American and New Zealand Facilities. Am. J. Ment. Defic..

[48] Rojahn J., Helsel W.J. (1991). The Aberrant Behavior Checklist with Children and Adolescents with Dual Diagnosis. J. Autism Dev. Disord..

[49] Brinkley J., Nations L., Abramson R.K., Hall A., Wright H.H., Gabriels R., Gilbert J.R., Pericak-Vance M.A., Cuccaro M.L. (2007). Factor Analysis of the Aberrant Behavior Checklist in Individuals with Autism Spectrum Disorders. J. Autism Dev. Disord..

[50] Kaat A.J., Lecavalier L., Aman M.G. (2014). Validity of the Aberrant Behavior Checklist in Children with Autism Spectrum Disorder. J. Autism Dev. Disord..

[51] Norris M., Aman M.G., Mazurek M.O., Scherr J.F., Butter E.M. (2019). Psychometric Characteristics of the Aberrant Behavior Checklist in a Well-Defined Sample of Youth with Autism Spectrum Disorder. Res. Autism Spectr. Disord..

[52] Muthén L.K., Muthén B.O. (1998–2017). Mplus User’s Guide-Eighth Edition.

[53] Finch W.H., Bolin J.E. (2017). Multilevel Modeling Using Mplus.

[54] Luke D.A. (2020). Multilevel Modeling.

[55] Woodman A.C., Smith L.E., Greenberg J.S., Mailick M.R. (2015). Change in Autism Symptoms and Maladaptive Behaviors in Adolescence and Adulthood: The Role of Positive Family Processes. J. Autism Dev. Disord..

[56] Brown W.H., Odom S.L. (1994). Strategies and Tactics for Promoting Generalization and Maintenance of Young Children’s Social Behavior. Res. Dev. Disabil..

[57] Carr E.G., Darcy M. (1990). Setting Generality of Peer Modeling in Children with Autism. J. Autism Dev. Disord..

[58] Jahr E., Eldevik S., Eikeseth S. (2000). Teaching Children with Autism to Initiate and Sustain Cooperative Play. Res. Dev. Disabil..

[59] Bauminger N., Solomon M., Rogers S.J. (2010). Externalizing and Internalizing Behaviors in ASD. Autism Res..

[60] Cibralic S., Kohlhoff J., Wallace N., McMahon C., Eapen V. (2019). A Systematic Review of Emotion Regulation in Children with Autism Spectrum Disorder. Res. Autism Spectr. Disord..

[61] Greenlee J.L., Stelter C.R., Piro-Gambetti B., Hartley S.L. (2021). Trajectories of Dysregulation in Children with Autism Spectrum Disorder. J. Clin. Child. Adolesc. Psychol..

[62] Kochanska G., Coy K.C., Murray K.T. (2001). The Development of Self-Regulation in the First Four Years of Life. Child. Dev..

[63] Olson S.L., Ip K.I., Gonzalez R., Beyers-Carlson E.E.A., Volling B.L. (2020). Development of Externalizing Symptoms across the Toddler Period: The Critical Role of Older Siblings. J. Fam. Psychol..

[64] Hastings R.P. (2003). Behavioral Adjustment of Siblings of Children with Autism Engaged in Applied Behavior Analysis Early Intervention Programs: The Moderating Role of Social Support. J. Autism Dev. Disord..

[65] Ross P., Cuskelly M. (2006). Adjustment, Sibling Problems and Coping Strategies of Brothers and Sisters of Children with Autistic Spectrum Disorder. J. Intellect. Dev. Disabil..

[66] Verté S., Roeyers H., Buysse A. (2003). Behavioural Problems, Social Competence and Self-Concept in Siblings of Children with Autism. Child. Care Health Dev..

[67] Hall H.R., Graff J.C. (2012). Maladaptive Behaviors of Children with Autism: Parent Support, Stress, and Coping. Issues Compr. Pediatr. Nurs..

[68] Smith L.E., Greenberg J.S., Seltzer M.M., Hong J. (2008). Symptoms and Behavior Problems of Adolescents and Adults With Autism: Effects of Mother–Child Relationship Quality, Warmth, and Praise. Am. J. Ment. Retard..

[69] Ganjiwale D., Ganjiwale J., Sharma B., Mishra B. (2016). Quality of Life and Coping Strategies of Caregivers of Children with Physical and Mental Disabilities. J. Fam. Med. Prim. Care.

[70] Gupta V.B. (2007). Comparison of Parenting Stress in Different Developmental Disabilities. J. Dev. Phys. Disabil..

[71] Greene R.W., Beszterczey S.K., Katzenstein T., Park K., Goring J. (2002). Are Students with ADHD More Stressful to Teach?: Patterns of Teacher Stress in an Elementary School Sample. J. Emot. Behav. Disord..

[72] Lecavalier L., Leone S., Wiltz J. (2006). The Impact of Behaviour Problems on Caregiver Stress in Young People with Autism Spectrum Disorders. J. Intellect. Disabil. Res..

[73] Cicirelli V.G. (1982). Sibling Influence Throughout the Lifespan. Sibling Relationships.

[74] El-Ghoroury N.H., Romanczyk R.G. (1999). Play Interactions of Family Members Towards Children with Autism. J. Autism Dev. Disord..

[75] McCauley J.B., Pickles A., Huerta M., Lord C. (2020). Defining Positive Outcomes in More and Less Cognitively Able Autistic Adults. Autism Res..

[76] Orsmond G.I., Seltzer M.M. (2000). Brothers and Sisters of Adults With Mental Retardation: Gendered Nature of the Sibling Relationship. Am. J. Ment. Retard..

