# Supplementary material for: Sibling Influences on Trajectories of Maladaptive Behaviors in Autism
Source: J Clin Med. 2022 Sep 12;11(18):5349. doi: 10.3390/jcm11185349 (PMC9502075; doi:10.3390/jcm11185349)
Supplement: Supplementary file 1 [file jcm-11-05349-s001.zip › jcm-1889128-supplementary.pdf]

Table S1. Teacher- and parent-reported ABC sample sizes across age groupings.

**Age (in years)**	**Teacher Report**		**Parent Report**	
	n	Datapoints	*n*	Datapoints
**9-10**	64	99	69	94
**11-12**	66	130	117	251
**13-14**	120	297	117	405
**15-16**	96	227	53	143
**17-18**	80	143	25	39

Note: *n* = number of unique participants during a specified age grouping; Datapoints = number of entries across all participants during a specified age grouping.
